# Comparative effects of non-steroidal anti-inflammatory drugs (NSAIDs) on blood pressure in patients with hypertension

**DOI:** 10.1186/1471-2261-12-93

**Published:** 2012-10-24

**Authors:** Hisham Aljadhey, Wanzhu Tu, Richard A Hansen, Susan J Blalock, D Craig Brater, Michael D Murray

**Affiliations:** 1 Medication Safety Research Chair, College of Pharmacy, King Saud University, Riyadh, Saudi Arabia; 2 Indiana University School of Medicine, Indianapolis, IN, USA; 3 Regenstrief Institute, Inc, Indianapolis, IN, USA; 4 Department of Pharmacy Care Systems, Harrison School of Pharmacy, Auburn University, Auburn, AL, USA; 5 Division of Pharmaceutical Outcomes & Policy, School of Pharmacy, University of North Carolina, Chapel Hill, NC, USA; 6 Purdue University College of Pharmacy, West Lafayette, IN, USA

**Keywords:** NSAIDs, Hypertension, Blood pressure, Propensity score

## Abstract

**Background:**

Nonsteroidal anti-inflammatory drugs (NSAIDs) may disrupt control of blood pressure in hypertensive patients and increase their risk of morbidity, mortality, and the costs of care. The objective of this study was to examine the association between incident use of NSAIDs and blood pressure in patients with hypertension.

**Methods:**

We conducted a retrospective cohort study of adult hypertensive patients to determine the effects of their first prescription for NSAID on systolic blood pressure and antihypertensive drug intensification. Data were collected from an electronic medical record serving an academic general medicine practice in Indianapolis, Indiana, USA. Using propensity scores to minimize bias, we matched a cohort of 1,340 users of NSAIDs with 1,340 users of acetaminophen. Propensity score models included covariates likely to affect blood pressure or the use of NSAIDs. The study outcomes were the mean systolic blood pressure measurement after starting NSAIDs and changes in antihypertensive therapy.

**Results:**

Compared to patients using acetaminophen, NSAID users had a 2 mmHg increase in systolic blood pressure (95% CI, 0.7 to 3.3). Ibuprofen was associated with a 3 mmHg increase in systolic blood pressure compared to naproxen (95% CI, 0.5 to 4.6), and a 5 mmHg increase compared to celecoxib (95% CI, 0.4 to 10). The systolic blood pressure increase was 3 mmHg in a subgroup of patients concomitantly prescribed angiotensin converting enzyme inhibitors or calcium channel blockers and 6 mmHg among those prescribed a beta-adrenergic blocker. Blood pressure changes in patients prescribed diuretics or multiple antihypertensives were not statistically significant.

**Conclusion:**

Compared to acetaminophen, incident use of NSAIDs, particularly ibuprofen, is associated with a small increase in systolic blood pressure in hypertensive patients. Effects in patients prescribed diuretics or multiple antihypertensives are negligible.

## Background

Cardiovascular diseases are the most common cause of death in the world [1], and uncontrolled hypertension is a harbinger of such poor outcomes. Seven million deaths worldwide each year are attributed to hypertension [2], and in the United States alone, about 73 million people, or one in three adults, have high blood pressure. Only 35% of these patients have adequate blood pressure control [3]. Chronic administration of medications that increase blood pressure is presumed to be among a variety of factors responsible for poorly controlled blood pressure that can lead to deleterious cardiovascular effects.

The effect of nonsteroidal anti-inflammatory drugs (NSAIDs) on the incidence of hypertension has been previously investigated [4-8]; however, little information is available about the magnitude of changes in blood pressure in populations of patients chronically taking antihypertensive medications. The effect of NSAIDs on blood pressure has been investigated in clinical trials [9-24] but not observational studies, which are more representative to real-world clinical settings. Therefore, we examined the association between NSAIDs and blood pressure in patients with hypertension who were taking antihypertensive medications. This observational study included incident users of NSAIDs and used propensity scores to minimize selection bias. Using these methods, we aimed to determine the comparative effects on systolic blood pressure of NSAIDs that are commonly used in primary care. Because acetaminophen is an oft-used alternative to NSAIDs, we determined the effects of NSAID relative to acetaminophen. Our objectives were to: (1) examine the association between NSAIDs and blood pressure compared to acetaminophen in patients with hypertension; (2) compare the effects of various NSAIDs on blood pressure in patients with hypertension; and, (3) examine changes in antihypertensive therapy after starting NSAIDs.

## Methods

### Design and subjects

This retrospective cohort study included adult patients who had received their first prescription for NSAID from the general medicine practice of Wishard Health Services in Indianapolis, Indiana. Wishard is a city-county health center affiliated with the Indiana University School of Medicine. The Regenstrief Medical Record System (RMRS) was used to identify eligible patients within Wishard and to collect data on relevant variables. The RMRS is an electronic medical record system that captures prescriptions, laboratory test results (including electrographic results), and other clinical data [25].

We included incident users in order to prevent under-ascertainment of adverse effects that occur early in treatment [26]. In this design only patients naïve to the study medication are included after the date of their first prescription (index date). Patients were eligible for inclusion if they had received a prescription for any NSAID between 1993 and 2006, were aged 18 years or older, and had a clinical diagnosis of hypertension on the index date. Patients were excluded if they had an active prescription for any NSAID during the year preceding the index date. Included patients had at least one measurement of sitting systolic blood pressure the year after the index date and no changes in their antihypertensive therapy until the measurement of blood pressure. Patients prescribed acetaminophen who met the same criteria described above formed a non-NSAID comparison group. Users of acetaminophen are similar to users of NSAIDs in most characteristics and comorbidities since acetaminophen is usually prescribed as a first line therapy for patients with osteoarthritis [27]. The study was approved by the Institution Review Boards at the University of North Carolina at Chapel Hill and Indiana University-Purdue University at Indianapolis.

### Data analysis

Systolic blood pressure measurements were collected one year before and one year after the date of the first prescription for NSAID or acetaminophen. Information was collected on relevant confounders at baseline. The end date of follow-up was defined as one year after the index date or 30 days after the last dispensed prescription, whichever came first.

Propensity score matching was used to prevent selection bias by balancing covariates between comparison groups. Propensity scores estimate the probability of each subject’s exposure to treatment A versus treatment B, based on measured covariates. The score combines all confounding covariates into a single composite factor. Mahalanobis metric matching without replacement was used since it produces a good balance in covariates between comparison groups [28,29]. A caliper of one-quarter of the standard deviation of the propensity score was used in the match. To assess the covariate balance, we used chi-square tests for categorical variables and t-tests for continuous variables. Separate propensity scores were estimated for each comparison. Propensity scores were calculated using SAS PROC LOGISTIC (SAS Institute, Inc., Cary, North Carolina).

After covariate balance, a multiple linear regression model was used to study the effect of NSAIDs on systolic blood pressure and changes in antihypertensive therapy. The primary independent variable was the use of NSAIDs. Covariates unbalanced after propensity score matching were included in the model. Also, the model included the time from index date until blood pressure measurement, since this time occurred after the index date it could not be included in the propensity score matching. Another advantage of using a regression model is that it reduces the standard error and, hence, improves the precision of the estimate [29].

We compared individual commonly used NSAIDs. Non-selective NSAIDs included ibuprofen and naproxen. The effects of naproxen and ibuprofen on blood pressure have not been compared in observational studies of patients using antihypertensive medications. Previous observational studies have not compared the effect of selective COX-2 inhibitors on blood pressure to non-selective NSAIDs. Since celecoxib is the only selective COX-2 inhibitor on the market, it was compared to the non-selective NSAIDs included in this study. We converted the odds ratios and related confidence intervals into relative risks using the conversion formula by Zhang and Yu [30]. We verified the model assumptions for linear regression analysis by examining the residuals of the model for signs of deviation from normality and unequal variability. As expected, the model assumptions were satisfied.

### Covariates

Based on previous literature, the models included covariates likely to affect blood pressure or the use of NSAIDs including age, race, gender, and baseline systolic blood pressure. Baseline systolic blood pressure was defined as the last measurement before the index date. The models controlled for the presence of the following diagnoses at the index date: rheumatoid arthritis, osteoarthritis, coronary artery disease or myocardial infarction, stroke (cerebrovascular accident or transient ischemic attack), arrhythmia, asthma or chronic obstructive pulmonary disease, renal insufficiency, cirrhosis with ascites, systemic lupus erythematosus, diabetes mellitus, and congestive heart failure. We controlled for the use of medications known to increase blood pressure including venlafaxine, a high dose of oral glucocorticoids, and the use of oral contraceptives.

To minimize bias introduced by variations in the time between baseline systolic blood pressure assessment and the index date and the time between the index date to the first systolic blood pressure, we included in the models indicator variables for these times and the index year. Variations in exposure to NSAID or acetaminophen were controlled by using the medication possession ratio (MPR) and the number of refills per month. The MPR assesses refill adherence and was calculated by dividing the sum of the days between the last refill and the next expected refill (i.e. days’ supply) by the number of days between the last refill and the next actual refill and then multiplied by 100. For each patient, an average MPR was calculated for the index drug. As a proxy for as-needed versus regular NSAID use, the number of refills per month was included in the model.

Sensitivity analyses were conducted to assess potential variations in exposure to the index drug. In one analysis, the model included the extent of exposure as the dose-MPR interaction. In the other, the analysis was restricted to only those patients who had a blood pressure measurement within 30 days of the index date. These results did not change our conclusions. Furthermore, we examined the dose effect of NSAIDs by stratifying patients into low and high dose groups. Patients who were prescribed less than 75% of the maximum daily dose listed in Facts and Comparisons [31] were included in the low dose category and those prescribed 75% or more were included in the high dose category.

Baseline use of antihypertensive medications was included as covariates in the models. Five groups of antihypertensive medications were formed: beta-adrenergic antagonists, calcium channel blockers (CCBs), diuretics, angiotensin-converting enzyme inhibitors (ACE-I) or angiotensin II receptor antagonists, and other antihypertensive medications. The MPR was used to control for adherence with antihypertensive medications.

### Endpoints

The outcomes of the study were the first and average systolic blood pressure measurements after starting NSAIDs, included as a continuous variable, and changes in antihypertensive therapy. Systolic blood pressure was assessed because it is associated with morbidity and mortality more so than diastolic blood pressure and is targeted in the treatment of hypertension [2]. The first blood pressure measurement following the drug index date was selected because physicians change antihypertensive therapy based on this measurement. Although, it was found in the same study population that one blood pressure reading has significant prognostic value [32], the results using an average of all blood pressure measurements were compared to the results when using only a single measurement. To prevent any potential effect of changing antihypertensive therapy on blood pressure, measurements of blood pressure were included only up to the date when the antihypertensive regimen was changed. The outcome was measured within one year of first NSAID prescription. Another analysis was conducted to investigate whether the increase in systolic blood pressure associated with NSAIDs is clinically important. A clinically important increase was defined as systolic blood pressure increase from baseline by at least 20 mmHg since the risk of mortality doubles for such an increase [2,33].

Changes in antihypertensive therapy post-index were considered as intensified when: (1) the dose of any currently prescribed antihypertensive medication was increased; or (2) the patient was started on a new antihypertensive medication from another class.

## Results

**Figure 1:**
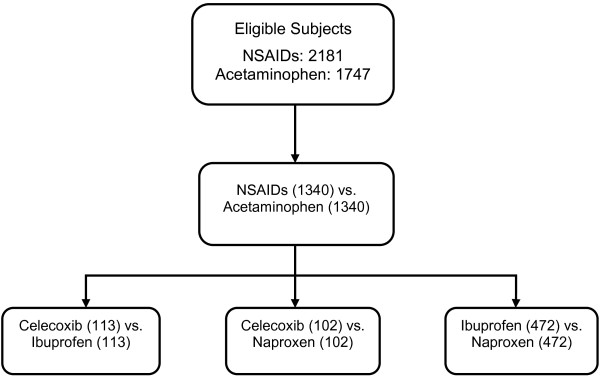
Study flow chart.

A total of 3,928 patients prescribed NSAIDs (n=2,181) or acetaminophen (n=1,747) met the inclusion criteria. Before matching on propensity score, many relevant baseline characteristics differed between the NSAID and acetaminophen cohorts (Table 1). Patients in the acetaminophen group were older and had a higher baseline mean systolic blood pressure compared to those in the NSAID group. They were also more likely to have renal insufficiency, congestive heart failure, diabetes, coronary artery disease or myocardial infarction, and stroke than those in the NSAIDs group Figure 1.

**Table 1:** Comparison of Covariate Balance between NSAIDs and Acetaminophen before and after propensity score matching

**Variable**	**Sample**	NSAIDs*	Acetaminophen*	P Value**	**Standardized Difference**	**Bias Reduction (%)**
Age (yrs) mean	Unmatched	55	60	<.001	−42.0	
	Matched	56	57	0.119	−6.0	86%
Gender:						
Female	Unmatched	70	70	0.687	1.3	
	Matched	72	70	0.157	5.5	−323%
Race:						
African American	Unmatched	58	63	0.003	−9.4	
	Matched	63	61	0.353	3.6	62%
Other	Unmatched	5	3	0.007	7.5	
	Matched	4	3	0.837	0.8	89%
Baseline systolic blood pressure (mmHg) mean	Unmatched	139	141	0.006	−8.8	
	Matched	140	140	0.949	−0.2	97%
Time from baseline SBP to index:						
≤ 7 days	Unmatched	53	65	<.001	−23.8	
	Matched	62	61	0.596	2.0	91%
> 7 days and ≤ 30 days	Unmatched	14	10	<.001	11.6	
	Matched	10	11	0.380	−3.4	71%
> 30 days	Unmatched	33	25	<.001	17.4	
	Matched	28	28	0.975	0.1	99%
Year of index date:						
1993 - 1996	Unmatched	50	58	<.001	−17.1	
	Matched	57	56	0.713	1.4	92%
1997-2002	Unmatched	39	40	0.477	−2.3	
	Matched	41	42	0.741	−1.3	44%
2003 - 2006	Unmatched	11	1	<.001	40.8	
	Matched	2	2	0.881	−0.6	99%
Diagnosis of:						
Osteoarthritis	Unmatched	22	23	0.768	−0.9	
	Matched	21	22	0.295	−4.0	−327%
Rheumatoid Arthritis	Unmatched	3	3	0.381	2.8	
	Matched	3	3	0.819	0.9	69%
Renal Insufficiency	Unmatched	3	8	<.001	−24.5	
	Matched	4	4	0.922	0.4	98%
Cirrhosis with Ascites	Unmatched	0.4	1	0.343	−3.0	
	Matched	1	1	0.807	−0.9	69%
Systemic Lupus Erythematosus	Unmatched	1	1	0.277	3.5	
	Matched	1	1	0.465	−2.8	20%
Diabetes	Unmatched	28	35	<.001	−14	
	Matched	28	31	0.124	−5.9	58%
Congestive Heart Failure	Unmatched	11	19	<.001	−20.9	
	Matched	14	14	0.651	−1.7	92%
Coronary Artery Disease or History of Myocardial Infarction	Unmatched	13	19	<.001	−16.0	
	Matched	14	15	0.266	−4.3	73%
Stroke	Unmatched	8	12	<.001	−15.4	
	Matched	9	9	0.784	−1.1	93%
Arrhythmia	Unmatched	1	2	0.055	−6.1	
	Matched	1	1	0.489	−2.7	56%
Asthma or Chronic Obstructive Pulmonary Disease	Unmatched	17	19	0.121	−5.0	
	Matched	18	19	0.420	−3.1	37%
Medications:						
ACE-I or Angiotensin II blocker	Unmatched	36	43	<.001	−15.7	
	Matched	37	38	0.594	−2.1	87%
Beta- Blocker	Unmatched	18	17	0.270	3.5	
	Matched	15	17	0.338	−3.7	−4%
Calcium Channel Blocker	Unmatched	34	38	0.003	−9.7	
	Matched	36	36	0.829	−0.8	91%
Diuretic	Unmatched	44	48	0.005	−9.0	
	Matched	46	45	0.921	0.4	96%
Other BP medications	Unmatched	7	12	<.001	−15.2	
	Matched	9	9	0.835	−0.8	95%
Oral high dose glucocorticoid***	Unmatched	1	1	0.068	−5.8	
	Matched	1	1	0.850	−0.7	87%
Oral Contraceptives	Unmatched	1	1	0.528	−2.0	
	Matched	1	1	0.998	0.0	100%
Venlafaxine	Unmatched	1	0.3	0.075	5.8	
	Matched	1	0.4	0.782	1.1	82%
Adherence to antihypertensive medications:						
MPR ≥ 80%	Unmatched	69	74	<.001	−10.6	
	Matched	72	70	0.320	3.8	64%
MPR < 80%	Unmatched	13	12	0.262	3.6	
	Matched	12	13	0.159	−5.4	−51%
Not using antihypertensives (reference group)	Unmatched	18	14	0.002	9.8	
	Matched	16	16	0.965	0.2	98%
Exposure to index drug:						
MPR > 80%	Unmatched	32	24	<.001	19.5	
	Matched	26	27	0.750	−1.2	94%
MPR 20–80 %	Unmatched	44	43	0.278	3.5	
	Matched	44	43	0.859	0.7	80%
MPR < 20%	Unmatched	23	34	<.001	−23.3	
	Matched	30	30	0.909	0.4	98%
Number of refills per month:						
≥ 1 refills	Unmatched	52	36	<.001	30.8	
	Matched	44	43	0.301	4.0	87%

* % unless indicated as mean. Because of rounding values may not add to 100 %.

** P-value of t-tests for continuous variables and chi-square tests for categorical variables.

*** High dose was defined as ≥10mg for prednisonse, ≥50mg for cortisone, and ≥1.5 mg for dexamethasone.

Standardized difference: 100 ( $\bar{\chi }$_treated_*–*$\bar{\chi }$_control_)/√ {(s^2^_treated_ + s^2^_control_)/2}. A positive value means the treated group is higher in % (or mean) compared to the control group and negative value means the control is higher than the treated.

Bias reduction (%) =1- {|Standardized difference _matched_|/| Standardized difference _unmatched_ |} x 100. A positive value means bias is reduced by propensity score matching and negative means bias increased.

ACE-I: Angiotensin converting enzyme inhibitor; MPR: Medication Possession Ratio; SBP: Systolic blood pressure. Unmatched: all patients before propensity score matching, N=3,928 (2,181 NSAIDs and 1,747 acetaminophen). Matched: only matched patients, N=2,680 (1,340 in each group).

A total of 1,340 patients prescribed NSAIDs were matched to the same number of patients who had been prescribed acetaminophen. Matching on propensity scores balanced all covariates between the two groups (Table 1). Compared to acetaminophen, NSAIDs were associated with a moderate mean increase in systolic blood pressure of 2 mmHg in patients with hypertension (95% confidence interval, 0.7 to 3.3) (Table 2). A prescription for NSAIDs was associated with a 3 mmHg increase in average systolic blood pressure in patients who were concurrently prescribed ACE-I or CCB, and a 6 mmHg increase in those prescribed beta-adrenergic blockers. However, no effect of NSAIDs on blood pressure was found in patients who were concurrently taking diuretics (Table 2). Changes in blood pressure were not associated with NSAIDs in patients prescribed various combinations of two or more antihypertensive medications (Table 2).

**Table 2:** Difference in systolic blood pressure between NSAIDs and acetaminophen after propensity score matching

**Sample**	Dependent Variable*	Estimate of SBP (mmHg)**	**95% Confidence Interval**
**All Patients (n=2,680)**	**First SBP**	**1.8**	**0.3 to 3.3**
	**Average SBP**	**2.0**	**0.7 to 3.3**
**ACE-I (n=768)**	**First SBP**	**2.8**	**−0.2 to 5.8**
	**Average SBP**	**2.8**	**0.2 to 5.4**
**CCB (n=804)**	**First SBP**	**2.5**	**−0.4 to 5.4**
	**Average SBP**	**3.2**	**0.6 to 5.7**
**BB (n=340)**	**First SBP**	**6.3**	**1.7 to 10.8**
	**Average SBP**	**5.5**	**1.4 to 9.6**
**Diuretics (n=1,022)**	**First SBP**	**0.2**	**−2.3 to 2.8**
	**Average SBP**	**1.3**	**−0.8 to 3.4**
**CCB & ACE-I (n=202)**	**First SBP**	**1.1**	**−5.6 to 7.8**
	**Average SBP**	**3.1**	**−2.8 to 8.9**
**CCB & BB (n=104)**	**First SBP**	**4.0**	**−5.0 to 13.0**
	**Average SBP**	**6.0**	**−2.0 to 14.1**
**CCB & diuretics (n=328)**	**First SBP**	**1.6**	**−3.3 to 6.5**
	**Average SBP**	**3.5**	**−0.8 to 7.9**
**ACE-I & BB (n=108)**	**First SBP**	**7.5**	**−1.0 to 16.0**
	**Average SBP**	**6.7**	**−1.1 to 14.5**
**ACE-I & diuretics (n=366)**	**First SBP**	**1.0**	**−3.4 to 5.5**
	**Average SBP**	**1.2**	**−2.7 to 5.1**
**BB & diuretics (n=156)**	**First SBP**	**3.8**	**−3.9 to 11.5**
	**Average SBP**	**4.2**	**−2.8 to 11.3**
**CCB & ACE-I & diuretics (n=100)**	**First SBP**	**1.5**	**−7.9 to 10.8**
	**Average SBP**	**3.8**	**−4.9 to 12.4**
**BB & ACE-I & diuretics (n=42)**	**First SBP**	**6.8**	**−9.4 to 22.9**
	**Average SBP**	**5.4**	**−10.3 to 21.1**

ACE-I: Angiotensin converting enzyme inhibitor; BB: Beta-blocker; CCB: Calcium channel blocker; SBP: Systolic blood pressure.

* First SBP is the first systolic blood pressure measurement after the index date. Average SBP is the average of all systolic blood pressure measurements after the index date and prior to any changes in the antihypertensive therapy.

** Estimate of SBP is the estimate difference between NSAIDs and acetaminophen after controlling for baseline SBP. A higher value means NSAIDs is associated with higher increase in systolic blood pressure compared to acetaminophen.

Based on their propensity scores, 472 patients from the naproxen group were matched to the same number of patients from the ibuprofen group. Matching on propensity scores resulted in balanced covariates between the two treatment groups. Compared to naproxen, ibuprofen was associated with a 2.5 mmHg increase in average systolic blood pressure (95% confidence interval, 0.5 to 4.6), and ibuprofen was associated with a clinically important increase in systolic blood pressure as defined above (relative risk, 1.47; 95% confidence interval, 1.09 to 1.96). The absolute risk of clinically important blood pressure increase in the ibuprofen group was 20.6% and in the naproxen group it was 14.6% and the calculated number needed to harm was twelve patients.

Ibuprofen was associated with a 5.9 mmHg increase in average systolic blood pressure in patients who were prescribed a beta-adrenergic blocker (95% confidence interval, 0.0 to 11.7; N=130) compared to naproxen (Table 3). A prescription for ibuprofen in patients who were prescribed various combinations of two or more antihypertensive medications was not associated with significant changes in systolic blood pressure (Table 3).

**Table 3:** Difference in systolic blood pressure between naproxen and ibuprofen after propensity score matching

**Sample**	Dependent Variable*	Estimate of SBP (mmHg)**	**95% Confidence Interval**
**All Patients (n=944)**	**First SBP**	**−2.0**	**−4.4 to 0.4**
	**Average SBP**	**−2.5**	**−4.6 to −0.5**
**ACE-I (n=276)**	**First SBP**	**0.7**	**−4.1 to 5.4**
	**Average SBP**	**−1.1**	**−5.3 to 3.0**
**CCB (n=268)**	**First SBP**	**−2.3**	**−6.8 to 2.2**
	**Average SBP**	**−2.2**	**−6.1 to 1.7**
**BB (n=130)**	**First SBP**	**−4.3**	**−10.6 to 2.0**
	**Average SBP**	**−5.9**	**−11.7 to −0.01**
**Diuretics (n=340)**	**First SBP**	**−3.2**	**−7.6 to 1.2**
	**Average SBP**	**−3.3**	**−7.0 to 0.5**
**CCB & ACE-I (n=60)**	**First SBP**	**0.4**	**−11.9 to 12.7**
	**Average SBP**	**−0.8**	**−11.7 to 10.1**
**CCB & BB (n=34)**	**First SBP**	**−4.5**	**−18.8 to 9.7**
	**Average SBP**	**−4.4**	**−18.3 to 9.6**
**CCB & diuretics (n=118)**	**First SBP**	**−3.2**	**−11.3 to 5.0**
	**Average SBP**	**−3.2**	**−9.5 to 3.2**
**ACE-I & BB (n=40)**	**First SBP**	**−5.8**	**−19.1 to 7.5**
	**Average SBP**	**−10.1**	**−23.0 to 2.8**
**ACE-I & diuretics (n=124)**	**First SBP**	**−3.3**	**−10.5 to 4.0**
	**Average SBP**	**−4.3**	**−11.0 to 2.5**
**BB & diuretics (n=70)**	**First SBP**	**−7.0**	**−16.3 to 2.4**
	**Average SBP**	**−5.4**	**−14.0 to 3.2**
**CCB & ACE-I & diuretics (n=28)**	**First SBP**	**−4.6**	**−22.9 to 13.8**
	**Average SBP**	**−0.5**	**−16.5 to 15.6**
**BB & ACE-I & diuretics (n=22)**	**First SBP**	**−7.4**	**−29.2 to 14.4**
	**Average SBP**	**−8.9**	**−30.8 to 13.0**

ACE-I: Angiotensin converting enzyme inhibitor; BB: Beta-blocker; CCB: Calcium channel blocker; SBP: Systolic blood pressure.

* First SBP is the first systolic blood pressure measurement after the index date. Average SBP is the average of all systolic blood pressure measurements after the index date and prior to any changes in the antihypertensive therapy.

** Estimate of SBP is the estimate difference between naproxen and ibuprofen after controlling for baseline SBP. A positive value means naproxen is associated with higher increase in systolic blood pressure compared to ibuprofen. A negative value means ibuprofen is associated with higher increase in systolic blood pressure compared to naproxen.

Dose effects of ibuprofen or naproxen on blood pressure were not statistically significant. A high dose of ibuprofen increased systolic blood pressure, 2.3 mmHg (95% confidence interval, – 1.3 to 5.1) and a high dose of naproxen slightly decreased in systolic blood pressure by – 3.3 mmHg (95% confidence interval, – 9.6 to 3.1).

Based on their propensity scores, 113 patients prescribed celecoxib were matched to 113 patients from the ibuprofen group. A prescription for ibuprofen was associated with a 5.2 mmHg increase in the mean systolic blood pressure compared to celecoxib (95% confidence interval, 0.4 to 10.0) (Table 4). Compared to ibuprofen or naproxen, celecoxib was not associated with a clinically important increase in systolic blood pressure.

**Table 4:** Difference in systolic blood pressure between celecoxib and ibuprofen or naproxen after propensity score matching

** Comparison**	Dependent Variable*	Estimate of SBP(mmHg)**	**95% Confidence Interval**
**Celecoxib vs. Ibuprofen (n=226)**	**First SBP**	**−5.4**	**−10.8 to 0.0**
	**Average SBP**	**−5.2**	**−10.0 to −0.4**
**Celecoxib vs. Naproxen (n=204)**	**First SBP**	**−0.3**	**−5.5 to 4.9**
	**Average SBP**	**−0.3**	**−5.1 to 4.5**

SBP: Systolic blood pressure.

* First SBP is the first systolic blood pressure measurement after the index date. Average SBP is the average of all systolic blood pressure measurements after the index date and prior to any changes in the antihypertensive therapy.

** Estimate of SBP is the estimate difference between celecoxib and the comparator drug (ibuprofen or naproxen) after controlling for baseline SBP. A positive value means celecoxib is associated with higher increase in systolic blood pressure compared to the comparator drug. A negative value means the comparator drug is associated with higher increase in systolic blood pressure compared to celecoxib.

When the outcome was defined as a change in antihypertensive therapy, 2,494 patients in the NSAID group were matched based on their propensity score to the same number of patients in the acetaminophen group. Compared to acetaminophen, receipt of NSAID was not associated with a change in antihypertensive therapy (odds ratio, 0.95; 95% confidence interval, 0.84 to 1.08; p = 0.4).

## Discussion

In the current study, patients receiving NSAIDs showed a 2 mmHg increase in systolic blood pressure compared to acetaminophen recipients. The systolic blood pressure increase was 3 mmHg in a sub-sample of those who were concomitantly prescribed ACE-I or CCB and 6 mmHg in those prescribed a beta-adrenergic blocker. Ibuprofen was associated with a systolic blood pressure increase, compared to both naproxen and celecoxib, of 3 and 5 mmHg, respectively. Despite these effects we did not detect significant changes in antihypertensive therapy in the NSAIDs users suggesting clinicians were not noticing these blood pressure changes that were admittedly subtle though sufficient to increase risk.

Similar to the current results, previous studies reported an up to 7 mmHg increase in blood pressure in patients who were stable on beta-adrenergic antagonists and had started NSAID therapy [34,35]. Interestingly, in our study the blood pressure increase associated with NSAIDs was greatest in patients prescribed a beta-adrenergic antagonist compared to other antihypertensive medications. The reason for this variation in blood pressure among antihypertensives could be related to the degree of prostaglandin (PGs) inhibition and the differences among these medications in their antihypertensive mechanisms. A proposed mechanism to explain this effect with beta-adrenergic antagonists is that inhibition of PGs by NSAIDs could increase sensitivity to the vasoconstrictor effects of sympathetic nervous system stimulation. Blocking beta receptors increases this sensitivity to the alpha sympathetic nervous system, resulting in abolishment of the blood pressure lowering effect of beta-adrenergic antagonists [36]. Further, some beta-adrenergic antagonists reduce the glomerular filtration rate [37]. In the long-term, this could increase the sensitivity to blood pressure increases by NSAIDs. This effect has important implications for those patients with heart failure and hypertension who have been prescribed beta-adrenergic antagonists.

The blood pressure increase we observed with NSAIDs in ACE-I users agrees with previous studies that reported a 5 to 10 mmHg increase in systolic blood pressure [13,18,19,23]. The inhibition of PGs by NSAIDs is proposed as the mechanism that explains the loss of the blood pressure lowering effect of ACE-I. Because PGs mediate the antihypertensive effect of ACE-I at least in part, inhibition of PGs by NSAIDs could disrupt the blood pressure control achieved by ACE-I [19,22,38]. These observations may be particularly important in patients with diabetes. Antihypertensive treatment is often intensified in patients with diabetes mellitus [39]; in addition, patients with diabetes mellitus who are diagnosed with hypertension are more likely to receive an ACE-I rather than other antihypertensive medications to preserve renal function. Therefore, it is important to monitor blood pressure closely in diabetic patients who are prescribed NSAIDs to ensure adequate blood pressure control.

No statistically significant changes in systolic blood pressure were associated with a prescription for NSAID in patients who were prescribed multiple antihypertensive medications. This can be explained by small sample size in some of these combinations. Because some of the combinations with beta-adrenergic blockers involved only small number of patients, it is possible that this study was not powered to detect small effects.

Similar to previous studies [10,11], the current study found no effect of NSAIDs on blood pressure in patients who were using diuretics. Current hypertension guidelines recommend starting patients on thiazide diuretics because they are associated with better clinical outcomes and lower mortality rates than other antihypertensive medications [2]. In addition, diuretics are often less expensive than other antihypertensive medications. The absence of an effect of NSAIDs is further reinforcement for use of diuretics to control blood pressure in patients who were using NSAIDs.

The results of the current study may have some clinical implications. Blood pressure is often poorly controlled in hypertensive patients [3], Our results raise the prospect that NSAID use contributes to that poor control found in numerous epidemiologic surveys. It is possible that more attention to the effects of NSAIDs on maintaining or achieving blood pressure control could lower morbidity and mortality and in so doing reduce health care costs [40]. For example, it was estimated that in the United States achieving or maintaining blood pressure control in users of selective COX-2 inhibitors would prevent more than 70,000 deaths from stroke and 60,000 others from coronary heart disease; such control would also result in direct health care cost savings of more than 3.8 billion dollars [40]. The small increase in systolic blood pressure associated with NSAIDs seen in this study may not affect a physician’s decision to change antihypertensive therapy. However, in the long-term, such an increase could be associated with significant comorbidity consequences. For example, decreasing systolic blood pressure by just 2 mmHg lowers stroke mortality by 10% and ischemic heart disease mortality by 7% [41]. Future studies are needed to assess the long term effect of such small increase in blood pressure.

This study has limitations that should be considered when interpreting the results. Patients included in this study came from a single health system and may not be representative of other practices. Hence, this study should be replicated in other clinical settings. Although propensity score matching balances many covariates at baseline, unobserved covariates could still differ between the groups. Bias is a threat to the validity of these results especially when comparing NSAID and acetaminophen groups. Acetaminophen has mild pressor effects that may have dampened the relative effects of NSAID [42]. Nonetheless, acetaminophen is often used as an alternative to NSAIDs and we therefore believed it was a reasonable non-NSAID comparator. Finally, several NSAIDs are available over the counter (OTC) as well as by prescription and this database captures only the use of prescription NSAIDs. However, because patients included in this study were provided with needed OTC NSAIDs through a prescription assistance program, it is less likely that they would have purchased additional OTC NSAIDs. Furthermore, sensitivity analysis research suggests that missing OTC drug exposure is not a significant source of bias [43].

In conclusion, compared to acetaminophen, incident use of NSAIDs (particularly ibuprofen) is associated with a small increase in systolic blood pressure in hypertensive patients. Effects in patients prescribed diuretics or multiple antihypertensives are negligible.

## Competing interest

The author(s) declare that they have no competing interests.

## Authors’ contribution

All authors contributed to the study idea, design, and methods. HA carried out the statistical analyses and drafted the manuscript. All authors contributed to the manuscript writing. All authors reviewed and edited the final version of the manuscript. All authors read and approved the final manuscript.

## Pre-publication history

The pre-publication history for this paper can be accessed here:

http://www.biomedcentral.com/1471-2261/12/93/prepub
